# The effectiveness of a dialogical family guidance intervention regarding child treatment response in families with a child with neurodevelopmental disorders

**DOI:** 10.1186/s40359-024-01706-9

**Published:** 2024-04-05

**Authors:** Diana Cavonius-Rintahaka, Mervi Roos, Anna Liisa Aho

**Affiliations:** 1 Faculty of Social Science, Nursing Science, University of Tampere, Tampere, Finland; 2 Neuropsychiatric Unit, Helsinki University Hospital (HUH), Child psychiatry, Helsinki, Finland

**Keywords:** Children, Dialogical family guidance, Neurodevelopmental disorders, Neuropsychiatric disorders, Parent strengths and difficulties questionnaire, Parents

## Abstract

**Background:**

Children with neurodevelopmental disorders (NDD) can have emotional and behavioral symptoms affecting not only the child, but the whole family. Since family members have a strong impact on each other, studies highlight the need to offer effective family interventions to strengthen the wellbeing of the family. The aim of the current study is to clarify whether there is a difference between parents` opinions regarding their child`s emotional and behavioral condition immediately after Dialogical Family Guidance (DFG) has ended and after a three and six month follow-up.

**Method:**

Fifty families with a child with NDD were randomized into two groups. Group 1 received DFG with an immediate starting point, and Group 2 received DFG after a three-month waiting period. Parent experiences of treatment response regarding their children`s emotional and behavioral symptoms were estimated before and after DFG using the parent version of the Strengths and Difficulties Questionnaire (SDQ-p) at baseline, and after three and six months. Additionally, comparisons between boys and girls, and the age of the child were analyzed.

**Results:**

The total difficulties score between Group 1 and Group 2 showed no difference immediately after DFG, or after three months. Regarding subdomains boys had more peer problems than girls, and at baseline, children between 3 and 6 years appeared to have more conduct problems than children between 7 and 13 years. Subdomain prosocial behavior increased statistically significantly during the study period in Group 1. Other SDQ-p subdomains remained constant in both groups between baseline and three and six month follow-up.

**Conclusions:**

The result does not show any differences between parents` opinions regarding their child immediately after or three months after DFG regarding SDQ-p total difficulties scores in either group. The difference between younger and older children regarding conduct problems at baseline, and the difference between boys and girls regarding peer problems is worth paying attention to in the clinical setting. Because of the small sample, it is not possible to draw relevant conclusions regarding the intervention`s effect regarding the child`s mental health dimensions, gender, or age. Nevertheless, Dialogical family Guidance represents one intervention that can be used.

**Trial registration:**

ClinicalTrials.gov NCT04892992 (retrospectively registered May 18th 2021).

## Background

Neurodevelopmental Disorders (NDDs) are a group of disorders involving neurological and psychiatric deficits, also described as neuropsychiatric disorders. NDDs have a starting point in early childhood and can include multiple disorders affecting learning, language, intellectual capacity, motor development issues such as coordination, or appear as attention deficit hyperactivity disorder (ADHD), autism spectrum disorder (ASD), tic-disorder, oppositional defiant disorder (ODD), or obsessive-compulsive disorder (OCD). Overlaps within these impairments are common, as well as the presence of supplemental impairments, usually called as comorbidities [1–4]. Common comorbidities can be for example ODD and behavior and anxiety disorders that result in difficulties to manage the complicated and difficult symptoms [5]. The prosocial dimension of social competence (for example empathy, cooperating skills) can also be affected, although children`s self-perception can be different compared to adults` experiences [6]. The adverse effects, symptoms, and the profile of NDDs may change especially during the childhood period, and Gillberg [7] presents these multiple NDD symptoms under a concept called ESSENCE (Early Symptomatic Syndromes Eliciting Neurodevelopmental Clinical Examinations).

Usually, NDDs run in the family, signifying a high heredity factor. Family studies show a strong familial incidence with NDDs, for example 80% in ASD [8–10] and 76% in ADHD [11]. This means that a child with an NDD in the family often implies that there is a major possibility that at least one another family member may also have an NDD, or symptoms of an NDD [12].

There is no doubt that NDD symptoms appear strongly among family members, and parent-child genetic similarity is a naturally influencing factor via parenting behavior. However, targeted work with children and parents has been shown to be effective in empowering parenting skills and improving the parent-child relationship [3, 4, 13–15].

Non-pharmacological methods are recommended for children in this target group. Training and interventions for children can target multiple different deficits under the NDD umbrella, and for example, cognitive training programs targeting executive function deficits offer acceptable and feasible interventions. These kinds of interventions can reduce problematic symptoms, improve executive function deficits, and potentially enhance social functioning in school aged children [16, 17]. Treatment planning for pre-school aged children (3–5 years old) can be complicated because their developmental and physiological differences can vary. The identification of symptoms and consideration of interventions can take place before or at the beginning of school-age. However, only little is known about non-pharmacological treatment options for pre-school children, compared to treatment options regarding school-aged children [18, 19].

Multimodal and psychosocial treatments offered to children with ASD and ADHD have shown promising results among all age-groups. Because of their individuality and developmentally appropriate approach, they have been found to be effective in reducing behavior problems. But it is not only the comorbid conditions or developmental level of the child that specifies the form of intervention, and family factors like economic conditions, ethnicity, and parental health like psychopathology and cognition are involved [20, 21].

NDD symptoms may affect all aspects of the child’s life. The symptoms and their impact change during the years, including for example academic difficulties, social skills, and parent-child relationships. The impact is among other things also dependent on the child`s age and the demands, support and understanding of their environment. An environment that is sensitive and aware of the individual needs of the child is vital. Targeted parenting advice and support is evident to optimize the development of the child`s self-esteem and long-term mental health development [22]. Parents have their own personal experiences of family life, which is known to have an impact on how the questionnaires are filled out and can result in more specific gender-related information [23]. Accordingly, this study is aimed to target both parents.

Children with NDD often pose a challenge to parents, causing increased stress and difficulties in raising these children consistently and with confidence as parents. The child`s impairments can have a negative effect on the child`s development, but they also bring difficulties to the parents` management of their child`s symptoms. A dysfunctional family system can also lead to negative effects on parent mental health. Symptoms such as heightened stress levels and feelings of anxiety or depression can follow as symptoms of parents` illbeing. High levels of parenting stress itself may also have negative effects on family functioning and relationship factors inside the family. But despite a knowledge of the association between child ADHD and parental stress, our understanding alone is not enough if we do not successfully develop and offer accurate family interventions [24].

The family environment naturally has a crucial role in children`s physical, psychological, and emotional development. Consideration of parent-child relationships, parental functioning, and family dynamics combined with the prevailing family climate can offer an insight into understanding the possible risks faced in families. But particularly, families with children with NDDs can struggle with day-to day problems to a much greater extent than other families [25, 26].

Although it is known that NDD symptoms “run in the family”, only little is known about how parenting and family systems operate and how they influence the family system. Thus, more effort is needed to better understand what works when it comes to supporting parents and children in family interventions [13]. Early family interventions play an important role in families with a child with NDDs, and increasing parents` understanding regarding their child with NDDs may reduce the risk of dysfunctional parenting and parental stress. This paper describes the use of Dialogical Family Guidance (DFG) in families having a child with NDDs. The assumption is that family interventions can promote positive parent practices and increase their understanding of their child`s behavior, which may in turn lead to improvements in the child`s behavior. However, further controlled studies concerning families with children with NDDs and tailored family interventions are needed to confirm this [25, 27].

An SDQ-screening of children can help the early detection of NDDs and identify groups at-risk. When using SDQ-p as a screening tool for NDDs, studies reveal that problems with conduct, hyperactivity, and low prosocial behavior can characteristically be seen as a comorbidity in ASD and ADHD in 6–12-year-old boys [28]. Also, a study by Björnsdotter et al. [29] report that the SDQ-questionnaire can be filled by primary health care workers, teachers or parents as an initial tool for detecting psychiatric problems used within the context of screening, assessment and evaluation of interventions. Particularly, younger children with ADHD appeared to have more hyperactivity-inattention symptoms and more peer problems, compared to older children. Their study also revealed a significant gender effect, where girls had more emotional and prosocial behavior problems compared to boys. Problems with conduct, hyperactivity/inattention, peer problems and total difficulties scores showed no gender differences. Differences between psychometric evaluations have been reported depending on country, geographical populations, culture, and the mother`s, father`s or teacher`s reporting regarding SDQ [23, 29, 30]. However, a study by Girela-Serrano [31] predicts that adolescents with ADHD are probably going to need mental health services as adults if they present more severe symptoms of hyperactivity/impulsivity and emotional dysregulation based on SDQ-p scores obtained during adolescence.

In our study, we used the SDQ-p questionnaire to compare parent experiences of possible changes in their child`s behavior. The aim of this study is to clarify whether there is a difference between parents` opinions regarding their child`s emotional and behavioral condition immediately after the DFG intervention has ended, and after a three-month follow-up. We also wanted to study if there is a difference between boys and girls, and whether the age of the child (4–6 years old and 7–13 years old) makes a difference regarding the SDQ-p total difficulties score or its subdomains.

The Psychiatric Ethical Committee of the Helsinki University Hospital approved the study (106/13/03/03/2012). Informed consent was obtained in writing from all the participants (legal guardians, parents to children) who took part in the study.

## Methods

### Participants

Families with children with NDDs were invited to participate in the study. The children were referred to an actual neuropsychiatric unit for assessment and to be given treatment plans by a multidisciplinary health-care team. The team included a child neurologist, child psychiatrist, pediatric nurses, occupational therapist, speech therapist, psychologists, and social workers. Also, the treatment of the child could be carried out at this unit, or outside the clinic more closely to the child’s home.

All of the families in this study have a child with NDDs, for example ADHD, ASD, tics, or/and OCD. The children were aged between 4 and 13 years old, and the main diagnosis was most often ADHD and/or ASD along with additional comorbidities. Parents required adequate Finnish language skills to participate and were required to be the biological parent or stepparent of the child.

The decision to include fifty families was made prior to starting the data collection, based on the approximate number of new children visiting the clinic during the study period (January 2016 and December 2018). Seventy-nine families met the inclusion criteria during the data collection period. Families that refused to take part in the study (*n* = 29) gave the following reasons: problems with time-schedules (*n* = 17), having a long journey to the clinic (*n* = 3), feeling they did not need or were not interested in DFG (*n* = 5), other policlinic visits coming up (*n* = 3), and language issues (*n* = 1). A total of thirty-five (*N* = 35) families completed all the phases of the study.

### Study design and data collection

**Fig. 1:**
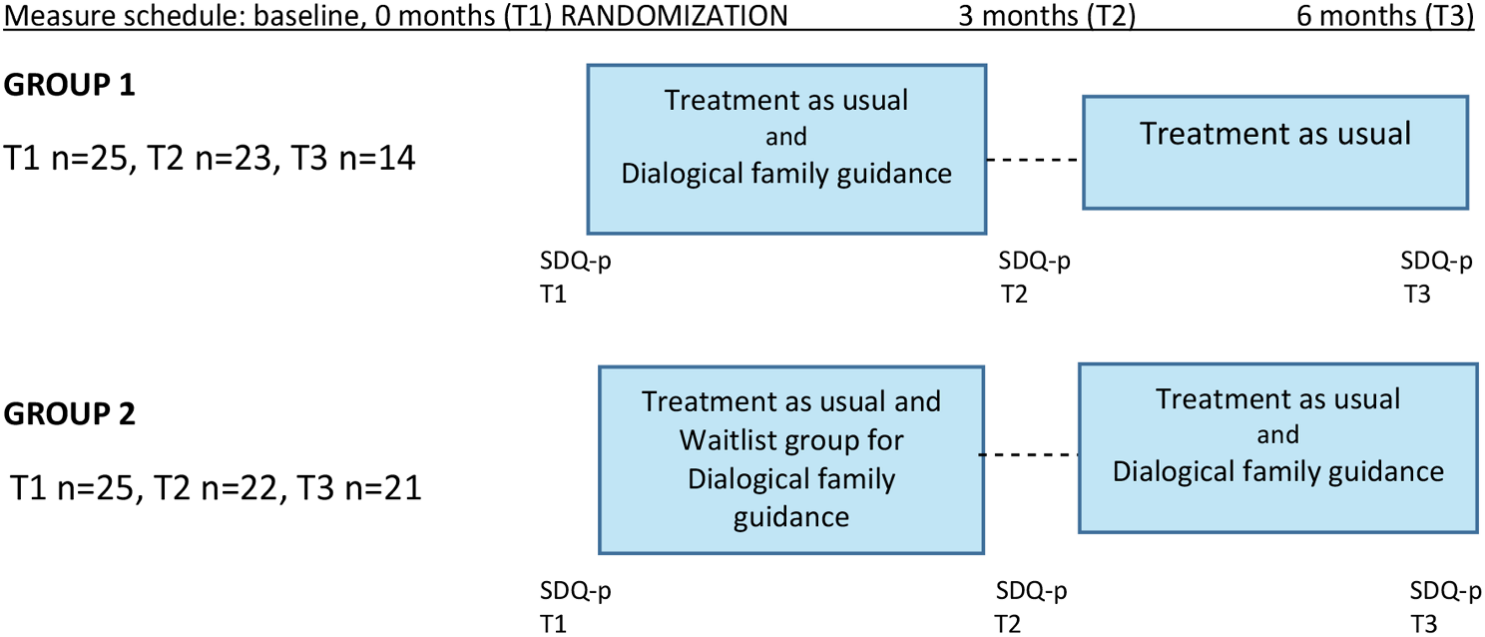
Study design and data collection

Parents, who`s child had a referral to the neuropsychiatric unit for the first time were invited to participate in this study when they arrived. Nurses delivered the invitation to parents, informing them about the study both orally and in writing. Families who gave their consent started by filling in the baseline questionnaires together. Families were randomized into two different groups (Group 1 and Group 2) through alternate allocation (Fig. 1). Parents could not choose what group they were placed in, and the answers given in their baseline questionnaires did not affect their group allocation. Randomization in this study were carried out by placing every second family in Group 1 and every second family in Group 2. The nurse informed the parents of what group they were placed in. Families who were placed in Group 1 were delivered DFG with a quick starting point (baseline) simultaneous with ordinary clinical treatment. Families in Group 2 were delivered DFG after three months of waiting, meaning three months after the baseline. Families in Group 2 were on waitlist for Dialogical Family Guidance, receiving ordinary clinical treatment during this time. This randomization process model was seen as an ethical one, giving all families who hoped to receive DFG a possibility to get it, although the starting point varied between families. (Fig. 1)

In addition to parent questionnaires, official medical reports were used to include the diagnosis of the children to form baseline demographics. The diagnoses of the children were defined by medical doctors and usually determined before (diagnosis already on the referral) or during the ongoing clinic visit to the neuropsychiatric unit. Any additional potential diagnoses received after the study period were not included.

### Questionnaire

The questionnaire included the demographic characteristics of parents and children, and the parental version of the Strengths and Difficulties Questionnaire (SDQ-p). The demographic data of parents and children was collected at the baseline stage of the study. The demographic data of parents included their gender, age, marital status, basic education, professional education, number of children, the quality of the relationship between parents, other members of the family with NDD or related diagnoses, parents’ self-reported health, and parental long-term illnesses. The demographic data of children included their gender, age, the child`s daytime activity, diagnoses mentioned on the medical referral, and the effect of the child`s NDD on his/her daily life according to the parents.

The parental version of the SDQ (SDQ-p) is aimed to briefly screen the emotional and behavioral condition of children and adolescents (SDQ-p 4–17 years), and to assess their strengths and difficulties. The SDQ-p includes 25 items, where 10 items reflect strengths, and 15 items reflect difficulties. Four subscales reflect difficulties (hyperactivity-inattention scale, emotional symptoms scale, conduct problem scale, and peer problem scale), and a total difficulties score can be formed by summing these subcategories. One subscale called the prosocial behavior scale reflects strengths. Each subscale consists of five items and the items are scored from 0 to 2 (0 = not true, never, rarely, 1 = somewhat, sometimes true, 2 = certainly true, often) [32–34, 35].

Decke et al. [36] report a SDQ-p score over 13 to be considered as indicative of a mental health problem. SDQ has been widely used as a screening instrument in community screening programmes to potentially increase the detection of child psychiatric disorders [37–39]. Studies report the psychometric properties of the SDQ-p in different countries and cultures, for example Portugal [40], Latvia [41], Finland [42, 43], Italy [44], and Norway [45].

### Procedures and intervention

Dialogical Family Guidance (DFG) is a family intervention tailored with help of a pilot study [46]. The DFG tailoring process focused specifically on creating a suitable family intervention for families with a child with NDD. Clinical experience from practice, theory-based research, and the findings of a pilot study form the basis of the DFG intervention. DFG is aimed to help all family members receive knowledge and gain an understanding of NDDs, providing a reflective space for family members to discuss their worries, thoughts, and feelings. The DFG includes three guidance areas: psychoeducation (didactic element), practical guidance for daily life (skill training), and emotional guidance. Emotional guidance includes family members` personal stories and unique experiences. Alongside the psychoeducation, the identification of the challenges and needs of all family members are seen as important [46–48].

A strong collaboration exists between the DFG-therapist and family members, who seek for effective parent strategies and skills to strengthen family members` relationships with each other. By using dialogue, it is possible to meet family members` individual goals, and share knowledge about the family system, family strengths, parenthood, the parents` relationship, family crises, and siblings´ reactions within the family. Usually, NDD have different impacts on family members. Open dialogue can help family members to become aware of the family dynamics and communication inside the family, and so initiate a mutual learning process. Although it is important to teach parents effecting parenting strategies, many parents may simply need the space to discuss their personal worries, thoughts, and feelings.

To become a certified DFG-therapist, you need to be a health care professional, with an own motivation and willingness regarding family-oriented working methods. Mainly registered nurses and social workers were attending the three-day training program before offering DFG to families participating to this study. These professionals had all at least three years’ experience from the field working with persons within neuropsychiatry. DFG educational program includes reflective discussions e.g. about family dynamics, family functioning, parenthood, siblings, dialogue. All the themes were connected to neuropsychiatry. Theoretical core topics e.g. parenthood and a child with NDDs, family systems theory, parental NDD, coping mechanisms in the family were offered side by side with the reflective discussions. Supervision and consultation were available for the DFG-therapists during the study proceedings by the researcher. The whole DFG-training program and DFG interventional details are published by Cavonius-Rintahaka et al. 2020. (47, 49–50).

The DFG collaborative working process generally includes six meetings (90 min per session) completed within a 3-month period. The initial session consists of dialogue between the DFG-therapist and parents, followed by completing an initial plan for the other upcoming five sessions. A session checklist is used to monitor content adherence, and a DFG-manual is provided to give structure during the DFG process. The manual contains suggested themes for each session (child`s development and demands in daily life, communication in the family, family resources and network, relation between siblings) [47].

All the families were receiving continuous ordinary clinical treatment for their child during the DFG intervention period. In the neuropsychiatric unit, care is focused mainly on the child including assessments to clarify their diagnosis, treatment planning, and sometimes the habilitation of the child is fulfilled within the unit. DFG was offered in addition to the ordinary clinical treatment. Usually, only the child with the referral gets attention at the clinic, and the other family members do not get any family interventions at that time.

In the study, every family participated in six sessions during their DFG, but the number of family members participating varied during the intervention, depending on the family’s needs and wishes.

### Statistical analysis

Statistical analysis was carried out using IBM SPSS Statistics for Windows, version 29 [51]. Differences between group 1 and group 2 in terms of parents’ demographic variables and children’s demographic variables were tested with Crosstabulation and Chi-square tests. Five subscales of SDQ-p were calculated by summing the items and dividing them by the number of items. The total difficulties score was calculated by summing the items that reflect difficulties. The reliability of all six sum variables was checked by Cronbach`s alpha. Five sum variables were normally distributed, except for Emotional problems at T1. The Mann-Whitney U-test were used to test differences between Group 1 and Group 2, between boys and girls, and between age groups in SDQ-p scores. The Wilcoxon Signed Ranks Test were used to test change in SDQ-p scores from baseline to three-month and six-month after intervention. The level of significance (p) was set as ≤ 0.05.

## Results

### Sociodemographic and participant data

Group 1 and Group 2 consisted at baseline of a total of 50 families. A total of 60 parents took part in this study. At baseline, 32 parents took part in Group 1 (12 fathers and 20 mothers), and 28 in Group 2 (8 fathers and 20 mothers). There were no statistically significant differences in background variables between these two groups. The mean age of the parents at baseline was 38 years (SD 5), and the mean age of parents in Group 1 was 37 (SD 5) and Group 2 was 39 (SD 6). In both groups, the mean number of children was 2 (SD 1, minimum 1 and maximum 6).

Twenty-nine parents were under 38 years of age (48%) and thirty-one parents more than 38 years of age (52%). Most parents were married/cohabiting (75%) and the rest reported not living together (25%). The education level of the parents was reported as university level for thirty-four parents (57%) and college level or under for twenty-six parents (43%).

The quality of the relationship between parents was reported to be excellent or good according to thirty-nine parents (65%) and moderate/poor or very poor according to twenty-one parents (35%). Other members of the family with neurodevelopmental disorders or diagnoses were reported to exist in seventeen families (28%), and not present in forty-three families (72%). Parent’s self-reported health was reported as very good or good by forty-six parents (77%) and moderate, poor, or very poor by fourteen parents (23%).

The characteristics of the children in Group 1 and Group 2 were quite similar (Table 1). The median age of all children was 6 (Q_1_ 5; Q_3_ 7): in Group 1 it was 6 (Q_1_ 5, Q_3_ 7) and in Group 2 it was 7 (Q_1_ 5, Q_3_ 8). The median age of the child when parents first became concerned regarding their neuropsychiatric problems was in Group 1: 3 (Q_1_ 2, Q_3_ 3) and in Group 2: 3 (Q_1_ 2, Q_3_ 4). The mean number of the child`s visits to the hospital or clinic before DFG were 5 (SD 3).

**Table 1:** Demographics of the children with NDD in families taking part in the study

	All children		Group 1		Group 2		
Background variables	%	n	%	n	%	n	p
**Gender**							0.247^1^
Girl	16.0	8	24.0	6	8.0	2	
Boy	84.0	42	76.0	19	92.0	23	
**Age (years)**							0.041^2^
< 7	62.0	31	76.0	19	48.0	12	
≥ 7	38.0	19	24.0	6	52.0	13	
**Child’s daytime activity**							0.037^2^
In daycare	66.0	33	80.0	20	52.0	13	
At school	34.9	17	20.0	5	48.0	12	
**Main diagnose of the child**							-
Attention-deficit/hyperactivity disorder	22.0	11	20.0	5	24.0	6	
Autism spectrum disorder	28.0	14	24.0	6	32.0	8	
Delayed milestone	20.0	10	32.0	8	8.0	2	
Speech and language disorders	12.0	6	12.0	3	12.0	3	
Specific learning disorder	8.0	4	-	-	16.0	4	
Other (motoric or psychiatric problems, unclear)	10.0	5	12.0	3	8.0	2	
**How the child’s NDD problems affect his/her daily life**							-
No symptom/hardly any symptoms	6.0	3	4.0	1	8.0	2	
Symptoms occasionally	10.0	5	4.0	1	16.0	4	
Symptoms often	40.0	20	48.0	12	32.0	8	
Symptoms disturbing all the time	44.0	22	44.0	11	44.0	11	

^1^Fisher’s exact test; ^2^Chi-Square test

### Effects on the child based on SDQ-p regarding the onset of DFG

There was no difference between the mean (SD) SDQ-p total difficulties scores between Group 1 and Group 2 at baseline: mean 17.68 (SD 5.39) in Group 1, and 16.68 (SD 5.18) in Group 2. Accordingly, the other SDQ-p domains were similar at baseline between Group 1 and Group 2. (Table 2)

When comparing Group 1 and Group 2 between baseline and six months, Group 1 finalized DFG three months earlier (3 months follow-up) and Group 2 finalized DFG at this stage. There was no difference in mean (SD) regarding SDQ-p total difficulties scores between Group 1 and Group 2 at six months: mean 18.93 (SD 5.90) in Group 1 and mean 18.71 (SD 5.57) in Group 2.

A statistical difference (*p* = 0.033) was found regarding the Prosocial subdomain between Group 1 and Group 2: mean 4.71 (SD 2.09) in Group 1 and mean 6.047 (SD 1.69) in Group 2. (Table 2)

Families in Group 1 received DFG with a quick starting point (baseline), and families in Group 2 were delivered DFG after three months waiting. The statistic result means that subdomain Prosocial behavior regarding children in Group 1 increased during the study period, indicating that children`s social skills increased. In Group 2 families were waiting three months for DFG to begin, the subdomain Prosocial behavior decreased during the study period, although these families also received DFG.

**Table 2:** Comparison between Group 1 and Group 2 at baseline, three months and six months based on SDQ-p

	Baseline (T1)			3 months (T2)			6 months (T3)		
	Group 1 (*n* = 25)	Group 2 (*n* = 25)		Group 1 (*n* = 23)	Group 2 (*n* = 22)		Group 1 (*n* = 14)	Group 2 (*n* = 21)	
SDQ-p	M (SD)	M (SD)	p	M (SD)	M (SD)	p	M (SD)	M (SD)	p
Total difficulties score	17.68 (5.39)	16.68 (5.18)	0.263	18.43 (4.50)	17.82 (6.33)	0.759	18.93 (5.90)	18.71 (5.57)	0.748
Emotional problems	2.24 (2.09)	2.72 (2.05)	0.297	2.91 (2.09)	3.32 (2.46)	0.614	2.07 (2.23)	3.19 (2.09)	0.154
Conduct problems	3.36 (1.89)	3.04 (2.15)	0.518	3.26 (1.66)	3.59 (2.40)	0.638	3.71 (1.86)	3.43 (2.16)	0.658
Hyperactivity, inattention	6.84 (2.90)	6.36 (2.34)	0.356	7.13 (2.34)	6.23 (2.62)	0.201	7.14 (2.03)	6.38 (2.56)	0.414
Peer problems	5.24 (2.18)	4.56 (2.26)	0.225	5.13 (2.51)	4.68 (2.147)	0.492	5.21 (2.86)	4.52 (2.02)	0.496
Prosocial behavior	5.16 (1.77)	5.24 (2.45)	0.589	5.00 (1.88)	5.64 (2.06)	0.375	4.71 (2.09)	6.047 (1.69)	**0.033**

Mann Whitney U-test

M = Mean

*T1 = baseline, T2 = 3 months, T3 = six months

### Comparison between genders at baseline, three and six months based on SDQ-p

At the baseline, the Peer problems subdomain scores showed statistically significant differences between boys and girls: mean 5.14 (SD 2.27) with boys, and mean 3.63 (SD 1.51) with girls (*p* = 0.036). After the three-month follow-up (T2), the Peer problems subdomain scores were mean 5.24 (SD 2.31) with boys, and mean 3.14 (SD 1.57) with girls (*p* = 0.020). At the six-month follow-up (T3) the Peer problems subdomain scores were mean 5.25 (SD 2.34) with boys, and mean 3.00 (SD 1.63) with girls (*p* = 0.028). (Table 3)

The result indicates that Peer problems increased regarding boys during the study period (T3 -T1). Regarding girls, Peer problems increased likewise a little during the study.

**Table 3:** Comparison between Boys and Girls at baseline, three and six months based on SDQ-p

	T1*			T2*			T3*		
	Boy (*n* = 42)	Girl (*n* = 8)		Boy (*n* = 38)	Girl (*n* = 7)		Boy (*n* = 28)	Girl (*n* = 7)	
SDQ-p	M (SD)	M (SD)	p	M (SD)	M (SD)	p	M (SD)	M (SD)	p
Total difficulties score	17.19 (5.55)	17.13 (3.56)	0.947	18.39 (5.76)	16.71 (2.81)	0.279	19.64 (5.61)	15.43 (4.58)	0.090
Emotional problems	2.43 (2.03)	2.75 (2.38)	0.707	3.03 (2.19)	3.57 (2.76)	0.646	2.82 (2.11)	2.43 (2.64)	0.614
Conduct problems	3.14 (2.08)	3.50 (1.69)	0.649	3.47 (2.20)	3.14 (0.69)	0.569	3.75 (2.08)	2.71 (1.60)	0.189
Hyperactivity, inattention	6.48 (2.54)	7.25 (3.11)	0.291	6.66 (2.34)	6.86 (3.44)	0.546	6.82 (2.14)	6.14 (3.24)	0.692
Peer problems	5.14 (2.27)	3.63 (1.51)	**0.036**	5.24 (2.31)	3.14 (1.57)	**0.020**	5.25 (2.34)	3.00 (1.63)	**0.028**
Prosocial behavior	4.98 (2.15)	6.38 (1.60)	0.079	5.21 (1.97)	5.86 (2.04)	0.357	5.39 (2.01)	6.00 (1.73)	0.680

Mann Whitney U-test

M = Mean

*T1 = baseline, T2 = 3 months, T3 = six months

### Comparison between children`s ages at baseline, three and six months based on SDQ-p

The total difficulties score at baseline was statistically significantly different in the two age groups. In the age group 4–6 years, the total difficulties SDQ-p score mean was 18.26 (SD 5.56), and in the age group 7–13 years, mean 15.42 (SD 4.30) (*p* = 0.038). (Table 4)

At the baseline, the Conduct problems subdomain showed a statistically significant difference. In the age group 4–6 years, the mean score was 3.68 (SD 1.96) and in the age group 7–13 years mean 2.42 (SD 1.89) (*p* = 0.028). (Table 4)

The total difficulties score at six months (T3) was similar in the two age groups. In the age group 4–6 years, the total difficulties SDQ-p score at six months mean was 19.545 (SD 5.43) and in the age group 7–13 years, mean 17.54 (SD 5.98). (Table 4)

The result indicates, at baseline (T1), younger children (4–6 years) had more Conduct problems compared to older children (7–13 years) being statistically significant. Subdomain Conduct problems decreased a little after three months (T2) and six months (T3) in the age group of 4–6 years. Regarding the older children (7–13 years), the subdomain Conduct problems increased a little between baseline and three months (T2), and six months (T3).

**Table 4:** Comparison between children`s Age at baseline, three and six months based on SDQ-p

	T1			T2			T3		
	Age 4–6 (*n* = 31)	Age 7–13 (*n* = 19)		Age 4–6 (*n* = 30)	Age 7–13 (*n* = 15)		Age 4–6 (*n* = 22)	Age 7–13 (*n* = 13)	
SDQ-p	M (SD)	M (SD)	p	M (SD)	M (SD)	p	M (SD)	M (SD)	p
Total difficulties score	18.26 (5.56)	15.42 (4.30)	**0.038**	19.00 (4.97)	16.40 (6.02)	0.135	19.545 (5.43)	17.54 (5.98)	0.364
Emotional problems	2.55 (1.96)	2.37 (2.27)	0.641	3.10 (2.12)	3.13 (2.59)	0.789	2.50 (2.30)	3.15 (2.30)	0.304
Conduct problems	3.68 (1.96)	2.42 (1.89)	**0.028**	3.73 (1.87)	2.80 (2.27)	0.138	3.91 (1.85)	2.92 (2.22)	0.157
Hyperactivity, inattention	5.74 (2.79)	6.37 (2.36)	0.479	6.97 (2.27)	6.13 (2.90)	0.347	7.046 (2.26)	6.08 (2.50)	0.300
Peer problems	5.29 (2.24)	4.26 (2.10)	0.219	5.20 (2.44)	4.33 (2.02)	0.238	5.09 (2.52)	4.31 (2.10)	0.407
Prosocial behavior	4.87 (1.78)	5.74 (2.54)	0.142	5.27 (1.87)	5.40 (2.23)	0.855	5.32 (1.99)	5.846 (1.91)	0.412

Mann Whitney U-test

*T1 = baseline, T2 = 3 months, T3 = six months

### Changes during the six-month study period based on SDQ-p

After three months compared to baseline (T2-T1), children’s emotional problems increased (*p* = 0.011). In Group 1 the change regarding emotional problems increased with statistical significance (Change: M = 0.61, SD = 1.31, *p* = 0.030). In Group 2, the change was smaller (M = 0.45, SD = 1.53, *p* = 0.151). The other SDQ-p subdomains remained constant in both groups between baseline (T1) and three-month follow-up (T2).

At three-month follow-up (T2) compared to baseline (T1) there were differences with boys concerning the subdomain of Emotional problems (M = 0.47, SD = 1.43, *p* = 0.035), where increases were seen in the age group of 4–6 years (M = 0.60, SD = 1.25, *p* = 0.011), but not in the age group of 7–13 years (M = 0.40, SD = 1.72, *p* = 0.359).

When comparing baseline (T1) to six-month follow-up (T3), the subdomain total difficulties score increased regarding boys (M = 1.54, SD = 3.33) and was statistically significant (*p* = 0.025). At three-month follow-up (T2) compared to baseline (T1) there were differences with age group 4–6 years concerning the subdomain Emotional problems (M = 0.60, SD = 1.25, *p* = 0.011).

## Discussion

The aim of this study was to clarify whether there is a difference between parents` opinions regarding their child`s emotional and behavioral condition immediately after DFG has ended and after a three-month follow-up compared to baseline. SDQ-p questionnaires were used to examine the DFG effect on the child. We also wanted to study if there is a difference between boys and girls, and whether the age of the child (4–6 years old and 7–13 years old) is significant. The effects of DFG from a parent perspective have been studied earlier [48] regarding family functioning, family health, and social support, and DFG was found to be effective. However, in this particular study, we wanted to focus on the child`s condition, even though it was from the parents` point of view.

This study did not show any difference in mean (SD) regarding SDQ-p total difficulties scores between Group 1 and Group 2 at six month follow-up. At this point, Group 1 had ended their DFG intervention three months earlier and Group 2 had ended DFG at that time. According to this result, there was no difference regarding the child`s condition according to parents, even though they had gone through the six sessions included in the DFG intervention. In this study, parents did not report any harmful nor unintended effects.

After received DFG intervention, parents in this study did not report any remarkable change in the behavior of their child and unfortunately, it is not possible to find an clear answer from this study regarding the effects connected to the onset of DFG. However, in previous research [48] regarding family health, functionality, and received social support before and after DFG, parents reported an increase of their experience of received social support and health of their family.

It can be assumed that in the NDD context, three and six month follow-up points are too short to capture any differences in children`s behavior, although DFG is supposed to help parents in everyday situations with their child in the home environment. It should be noticed that the children of the parents in this study were receiving hospital care at a university clinic at this time. This usually means that before the University clinic visits the child has been subject to often time-consuming clinical visits within their process of basic care before the University clinic visit is made available. At this stage, many of these parents are burdened and affected by long-time stress, and so positive changes can be difficult to discern during a short time.

Several studies [21, 22, 27] reinforce that guidance to parents can minimize the child`s behavioral symptoms (e.g., oppositional behavior), and so enhance parental confidence and reduce their stress. Alongside the positive effects on the behavior of the child, parent training can result in parents having a more positive attitude and constructing methods to handle their child [21, 22, 27, 52].

When looking at the results in this study regarding the subdomains included in the SDQ-p questionnaire, there is a statistical difference to be found between the scores reported in Group 1 and Group 2 regarding the Prosocial-domain, indicating strengthened positive social talents in Group 1 after DFG. This can be seen as a positive sign, although a longer follow-up and larger dataset is needed to make any firm conclusions. When comparing boys and girls, boys seemed to have more peer problems than girls and during the study period (six months) it appeared to increase. This is worth giving attention in clinical setting, although in this study we have only a short follow-up time to report. Also, the subdomain conduct problems at baseline seemed to be more obvious regarding younger children (4–6 years), compared to older children (7–13 years) in this study, meaning that younger children with NDDs can be especially demanding with their family members and friends and most likely affecting dynamics in the family and quality of relationships. At the same time, treatment planning for preschool aged children is reported to be demanding because their developmental differences [18, 19]. Additionally, younger children do not have diagnoses in early age and the awareness of NDDs among parents is naturally not yet sufficient at that time.

In this study, we got the parent perspective on their children’s social skills, but the study by Vuori et al. [6] reports that informant discrepancy can appear between parents and children. Accordingly, future research should offer children themselves an opportunity to complete the questionnaires from their own perspective. Additionally, other people such as teachers, grandparents or personnel in day care centers could offer complementary insights and perspectives regarding child behavior evaluations.

Due to the small sample size of this study, no comparisons based on gender or age can be made. However, according to Borg et al. [43], 4-9-year old Finnish children do show gender-based differences relating to SDQ-scores, where boys have higher scores meaning more health-related symptoms (total difficulties scores are reported as 9/10– borderline, and 11/12– abnormal).

In a study by Koskelainen et al. [42] that tested the SDQ-p questionnaire among primary and secondary school children, boys had higher total difficulty scores than girls in primary school. As been noticed in many reports regarding SDQ, higher scores indicate more behavioral difficulties, except for the prosocial subdomain where higher scores indicate higher resources [31, 32, 34, 37, 42]. It is notable that the boys and girls in our study has significantly high scores, verifying that at baseline, these children already have quite severe disorders and disturbances. Although the results obtained after the DFG intervention and the show no change when looking at the SDQ-p scores, some decrease of behavioral problems could still be possible. As previously mentioned, it could be that the follow-up time was too short for the parents to recognize positive changes in their child, but when the child has NDDs, then changes towards the better are often time and patience consuming.

### Limitations

Mother and father reports of SDQ-p are not examined separately in this study. This may give an incomplete picture of parents’ views on the child’s condition, as earlier studies acknowledge that father involvement is usually minor compared to that of mothers. Obtaining separate father and mother reports can be of importance to highlight the difference between parents, and is recommended when studying parents’ opinions. It is also acknowledged that informant combination can have an impact on concordance and estimation regarding the child’s symptoms [30, 44]. 

The number of respondents reduced during the study period, and not all of the parents who attended at baseline continued on to the six-month assessment point. Therefore, these findings must be seen only as preliminary, and further studies are needed.

The Cronbach´s alpha value for the total difficulties score was 0.59–0.63, and for the subscales between 0.54 and 0.76. These values can be considered as low, but are in line with other studies, and may relate to the small volume of data used in this study. Another perspective on this low Cronbach`s alpha is that the SDQ-p questionnaire was used as a data collection questionnaire, in order to measure the effectiveness of the DFG intervention and changes regarding child behavior. While the SDQ-p questionnaire is used and suited to clinical purposes, another questionnaire may have been more suitable for use in this study. The internal consistency of different SDQ scales has been analyzed with Cronbach’s alpha in a study by Koskelainen et al. [42], who report a total difficulties score of 0.71, and subscale scores in the range of 0.63–0.86. The Cronbach`s alpha in the study of Bezborodovs et al. [41] was scored as 0.786 for total difficulties and between 0.71 and 0.56 for the subscales.

Only parent reports based on the parents’ opinions were used in this study, and any further examinations by health care professionals and their opinions were not included. The results are therefore only the opinions of the parents who have taken part of this study and reflect their experiences of their children. Thus, it is possible that another parent sample could include different answers, and so yield different results.

As families received ordinary treatment alongside DFG, it meant discussions with professionals mainly connected to the assessment of their child. In practice, the health care needed for parents` own child was present and available for e.g. their questions during the study period. This most probably affected parents´ experiences about receiving support and help to their child, although, the rest of the family were not in focus during “treatment as usual” period. It was only during the DFG intervention, when siblings and parents received personal attention regarding issues concerning themselves or/and e.g. family dynamics involved. It is therefore important to give notice to circumstances of participants in this study. This is a limitation regarding this study when effectivity of DFG is discussed and evaluated.

With these limitations in mind, it is noted that the results for girls and boys are reported separately, which is of importance regarding the knowledge needed in clinical development processes. Also, the children’s age has been considered when conducting the study and also when reporting the results. However, in this study we could not see any differences relating to the gender or age of the children.

The power analysis was not made because of the small sample size, which can be seen as a limitation regarding the results. Most children in this study were already at baseline according to SDQ-p in high-risk category having behavioral difficulties of more severe kind. The sample can therefore be seen as more typical within university hospital care, but as not typical compared to primary health care population level.

Although, many limitations exist, this study offers new knowledge to professionals working with families in this target group. This study offers clinically relevant aspects involved when dealing with families with a child with NDD. Above all, the importance of involving all family members in the interventions is crucial.

## Conclusions

SDQ-screening of children has been tested in many studies and is regarded as an effective way to detect children’s early-stage psychopathological difficulties, emotional disturbances, and hyperactivity demands. This study addresses child symptom aspects that are familiar and common under the NDD umbrella and highlights the mental and peer relationship issues that are related to NDD symptoms. The results of this study contribute further knowledge needed when tailoring support and treatment for children with NDDs and their families. Although, this study could not demonstrate clear effect of the DFG intervention on children’s mental health dimensions, Dialogical Family Guidance remains a possible intervention to be used in the given context.

## Data Availability

The original data analyzed in this current study are not publicly available due to reasons of ethical privacy. Other data included in the study are available from the authors upon reasonable request.

## Abbreviations

DFG: Dialogical Family Guidance
NDD: Neurodevelopmental Disorders
ASD: Autism spectrum disorder
ADHD: Attention Deficit Hyperactivity Disorder
OCD: Obsessive Compulsive Disorder
